# Optimizing neutralization strategies for microbial testing of non-sterile pharmaceutical finished products with challenging method suitability

**DOI:** 10.1186/s12866-025-04456-9

**Published:** 2025-11-15

**Authors:** Ahmed Eldemerdash, Radwa Ewaisha, Mustafa Alseqely, Michael G. Shehat

**Affiliations:** 1 Borg Pharmaceutical Industries, New Borg El Arab, Alexandria, Egypt; 2 Department of Microbiology and Immunology, Faculty of Pharmacy, Alexandria University, 1 Khartoum Square, Azarita Alexandria, Egypt

**Keywords:** Microbial testing, Non-sterile pharmaceutical products, Pharmaceutical microbiology, Neutralization strategies, Microbiological quality control, Microbial recovery, USP

## Abstract

**Background:**

Method suitability for microbial limit tests during microbiological quality control (QC) can be a complicated process that requires multiple steps of optimization and plays a central role in ensuring reliable QC results. When antimicrobial activity of a given product cannot be neutralized, it is assumed that the inhibited microorganism is not present in the product, as specified by the U.S. pharmacopeia. This can potentially lead to contaminants that can multiply during storage or use, resulting in potential health risks or even death.

**Methods:**

Method suitability testing was performed on a total of 133 pharmaceutical finished products as part of microbiological QC testing performed prior to product marketing. For total microbial count, depending on the pharmaceutical dosage form, testing included sequential trials of different dilution factors up to 1:200, the addition of 1–5% tween (polysorbate) 80, 0.7% lecithin, and/or filtration using different membrane filter types until the optimal method was selected for each product. Method suitability for microbial limit tests as well as for absence of specific pathogens was verified using a range of bacteria and fungi including *Burkholderia cepacia*.

**Results:**

There was an acceptable microbial recovery of at least 84% for all standard strains with all neutralization methods, demonstrating minimal to no toxicity. Forty of 133 finished products required multiple steps of optimization. Of these, 18 were neutralized through 1:10 dilution with diluent warming. Another 8 had no inherent antimicrobial activity to their API and were neutralized through dilution and the addition of tween 80. Neutralization of the remaining 13 products (mostly antimicrobial drugs) was achieved through variations of different dilution factors and filtration with different membrane filter types with multiple rinsing steps.

**Conclusion:**

Our results provide detailed protocols that can help guide method suitability testing of finished pharmaceutical products for which neutralization proves challenging. We also include testing for *B. cepacia* complex in aqueous dosage form, often overlooked in the QC process. Since the current QC practice mostly assumes that these products do not require further testing, similar studies are especially needed to ensure the safety of finished pharmaceutical products during marketing, storage, and use.

## Background

Microbiological quality control (QC) of pharmaceuticals ensures quality and safety of pharmaceutical products by testing for microbial contaminants that may have been introduced before or during the manufacturing process. Microbial contaminants in non-sterile finished products beyond the allowed limits can multiply during storage or use of the pharmaceutical product, resulting in potential health risks or even death [1]. Pharmaceutical manufacturing companies adhere to strict guidelines issued by their respective governments, such as those described in the United States Pharmacopeia (USP), European Pharmacopeia (EP), and Japanese Pharmacopeia (JP).

Non-sterile pharmaceutical preparations must pass the appropriate microbial limit tests according to compendial acceptance criteria before they can be released to the market. For example, the total aerobic microbial count (TAMC) acceptance criterion in finished oral non-aqueous preparations is 10³ CFU/g, while the total combined yeast and mold count (TYMC) acceptance criterion is 10² CFU/g. Similarly, in finished oral aqueous preparations, the acceptance criteria for TAMC and TYMC are 10² CFU/mL, and 10 CFU/mL, respectively [2]. In addition, specific pathogens must be absent from certain types of pharmaceutical finished products. For instance, *Escherichia coli* must be absent from preparations intended for oral use while *Staphylococcus aureus*, and *Pseudomonas aeruginosa* should be absent from those intended for cutaneous use [2] (Table 1).

**Table 1:** Acceptance criteria of different pharmaceutical dosage forms specified by the U.S. Pharmacopeia

Dosage form and intended use	Total Aerobic Microbial Count (TAMC) (cfu/g or cfu/mL)	Total Yeast and Mold Count (TYMC) (cfu/g or cfu/mL)	Specific pathogens that need to be absent (in 1 g or 1 mL)
Nonaqueous preparations for oral use	10^3^	10^2^	*E. coli*
Aqueous preparations for oral use	10^2^	10^1^	*E. coli* and *B. cepacia*
Rectal use	10^3^	10^2^	N/A
Oromucosal use	10^2^	10^1^	*S. aureus*,* P. aeruginosa*, and *B. cepacia*
Gingival use	10^2^	10^1^	*S. aureus*,* P. aeruginosa*, and *B. cepacia*
Cutaneous use	10^2^	10^1^	*S. aureus*,* P. aeruginosa*, and *B. cepacia*
Nasal use	10^2^	10^1^	*S. aureus*,* P. aeruginosa*, and *B. cepacia*
Vaginal use	10^2^	10^1^	*S. aureus*, *P. aeruginosa*, and *C. albicans*
Dietary supplements	10^3^	10^2^	*E. coli*
Transdermal patches (limits for one patch including adhesive layer and backing)	10^2^	10^1^	*S. aureus* and *P. aeruginosa*
Inhalation use (special requirements apply to liquid preparations for nebulization)	10^2^	10^1^	*S. aureus*,* P. aeruginosa*,* B. cepacia*, and bile-tolerant Gram-negative bacteria

Method suitability for microbial limit tests is a critical and often complex process that plays a central role in ensuring reliable QC results. Method suitability testing evaluates residual antimicrobial activity of the product being tested, to ensure absence of any inhibitory effects on the growth of microorganisms under the conditions of the test [3]. The goal is to establish a method of testing for each raw material or finished product that neutralizes any antimicrobial activity, allowing the expected growth of control microorganisms and ensuring a method that can test for the organism in the presence of the product [4]. Antimicrobial activity of a finished pharmaceutical product may be due to active pharmaceutical ingredients (API) with antimicrobial properties, added preservatives or less commonly other excipients.

Test procedure includes the neutralization of antimicrobial activity using either dilution, chemical inhibition, membrane filtration, or a combination of one or more of these methods. This is followed by evaluating the ability of low inocula (usually < 100 CFU) of standard microorganisms to grow under the conditions of the neutralization method. Standard microorganisms specified by the USP are *Staphylococcus aureus*, *Bacillus subtilis*, *Pseudomonas aeruginosa*, *Candida albicans*, and *Aspergillus brasiliensis* [5]. According to the USP, if the antimicrobial activity of a given product cannot be neutralized during testing for a microorganism that should be absent or limited, then it is to be assumed that the inhibited microorganism is absent or limited in the finished product and that the product is not likely to be contaminated with the given species of the microorganism [3]. This assumption can potentially lead to contaminants in the marketed product that should either be absent or not exceed a certain count, especially in products for which the antimicrobial activity is difficult to neutralize.

Here, we describe detailed protocols for neutralization of the antimicrobial activity of products for which method suitability testing was challenging. These include protocols for both microbial enumeration and testing for specific organisms. This study was conducted as part of a large method suitability screening process for various pharmaceutical finished products. Of these, finished products that were more challenging and for which more than one neutralization method had to be tested are reported in this study. We describe the protocol followed to select the best neutralization method and discuss some of the challenges and potential solutions.

## Methods

### Microbiological QC assays, standard strains, and culture media

This analysis was part of microbiological quality control testing performed prior to product marketing. This was a study performed at Borg Pharmaceutical Industries between September 2022 and June 2025. Method suitability testing was performed on a total of 133 pharmaceutical finished products. Microbiological assays performed included total aerobic microbial count (TAMC), total combined yeast and mold count (TYMC), and absence of specific pathogens. Microbiological quality of finished products was assessed according to the criteria specified by the U.S. pharmacopeia (Table 1). TAMC is defined as the number of colony forming units (CFU) found using Soybean–Casein Digest Agar (SCDA; also known as tryptone soy agar; TSA). Fungal colonies detected on this medium were counted as part of TAMC. TYMC is defined as the number of CFU found on Sabouraud dextrose agar (SDA). Bacterial colonies detected on this medium were counted as part of TYMC. For each test, a sufficient volume of the microbial suspension that contained an inoculum of not more than 100 CFU was added to the product prepared as directed with the attempted neutralization methods as described below and to a control (with no test material). The volume of the inoculum did not exceed 1% of the diluted material volume. All tests were performed at least in duplicate and means were calculated and reported.

The following standard strains were used for testing microbial recovery – *Staphylococcus aureus* (ATCC 6538), *Escherichia coli* (ATCC 8739), *Pseudomonas aeruginosa* (ATCC 9027), *Aspergillus brasiliensis* (ATCC 16404), *Burkholderia cepacia* complex (ATCC 25416) and *Candida albicans* (ATCC 10231). Tryptone soy medium was used for total microbial growth (TAMC test), and Sabouraud dextrose medium for fungi (TYMC test). For testing for specific organisms, mannitol salt agar was used for *S. aureus*, cetrimide agar for *Pseudomonas aeruginosa*, and *Burkholderia cepacia* selective agar (BCSA) for *Burkholderia cepacia* complex.

### Inoculum preparation

McFarland standards were used to standardize the approximate number of microorganisms in a liquid suspension by comparing the turbidity of the test suspension with that of the 0.5 McFarland Standard. Microbial suspensions were adjusted to the same turbidity of the McFarland standard. Serial ten-fold dilutions were then prepared, and a plate count of the dilutions was performed. This allowed verification of the accuracy of the McFarland Standard and ensured that the suspension gave a representative colony count. That was done either by the colony suspension method or the growth methods. For the colony suspension method, the inoculum was prepared by suspending isolated colonies selected from an 18 to 24-hour agar plate (nonselective medium) in buffered sodium chloride peptone solution, or saline. The suspension was adjusted to achieve turbidity equivalent to a 0.5 McFarland standard. To perform this step accurately, a spectrophotometer device was used by adjusting transmittance at 580 nm. Alternatively, the growth method was used when colony growth was difficult to suspend directly, and a smooth suspension could not be achieved. The growth method was performed by transferring at least 3–5 well-isolated colonies of the same morphological type from an agar plate into a tube containing 4 to 5 mL of tryptic soy broth. The broth culture was incubated at 35 ± 2 °C for yeast and bacterial cultures or at 20–25 °C for *Aspergillus brasiliensis* until it achieved or exceeded the turbidity of the 0.5 McFarland standard. The turbidity of the actively growing broth culture was adjusted using a spectrophotometer with sterile saline, buffered sodium chloride peptone solution, or broth to achieve a turbidity equivalent to that of a 0.5 McFarland standard. To suspend *Aspergillus brasiliensis* spores, 0.05% of polysorbate 80 was added to sterile saline or sterile buffered sodium chloride peptone solution. For microbial count validation of the prepared microbial suspension for each microorganism, serial dilution of the initial suspension (which is equivalent to 0.5 McFarland standard) was performed, then plated on TSA agar. Finally, the initial count was calculated based on the dilution factor and the procedure was repeated at least three times to ensure reproducibility of the initial count. The count was then used to calculate the volume and the serial dilution needed to achieve the desired number of microorganisms used for each experiment.

### Optimization of neutralization methods

**Fig. 1:**
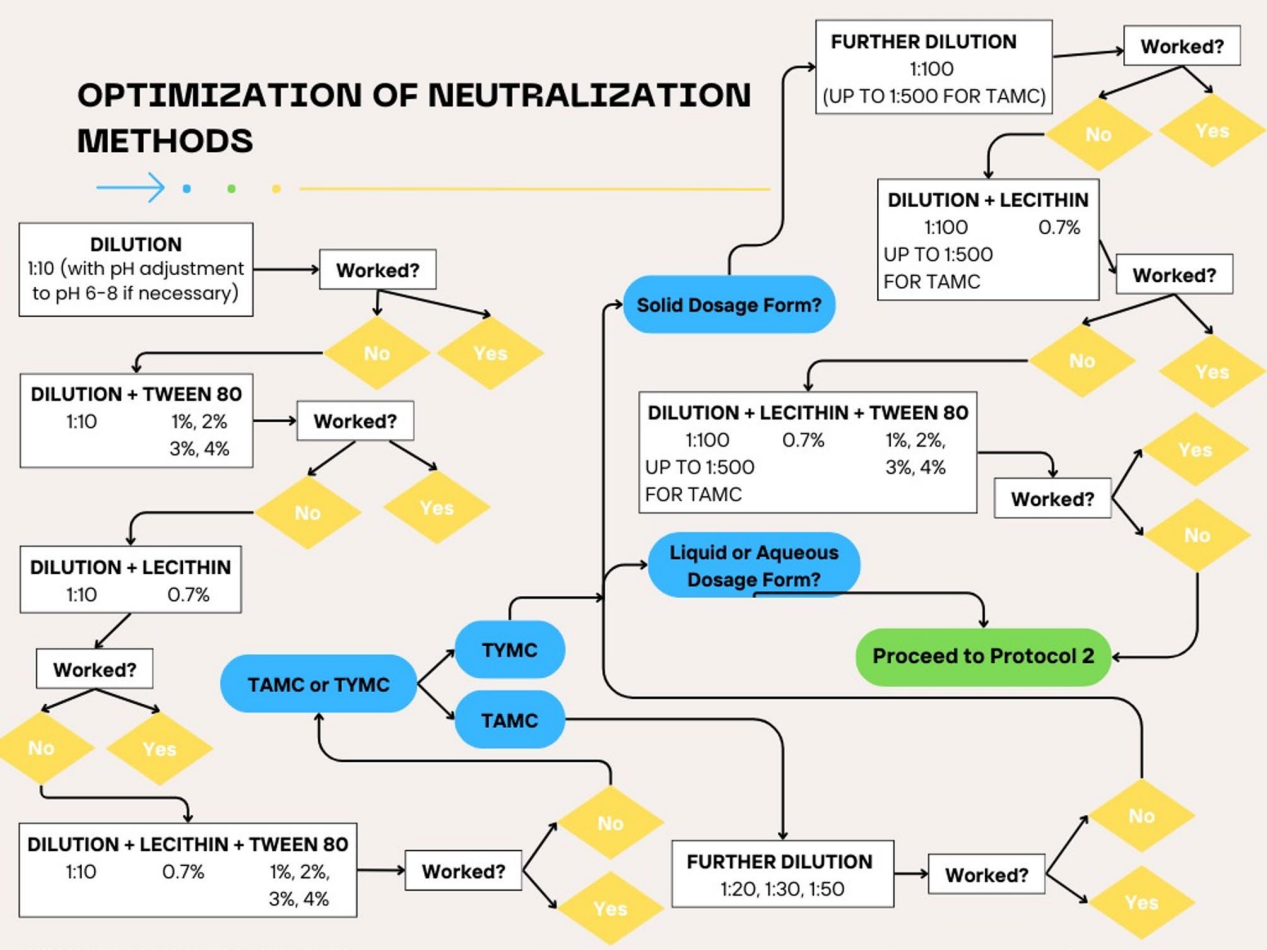
Protocol 1 followed for the optimization of neutralization methods for microbial enumeration of finished products using the pour plate method

**Fig. 2:**
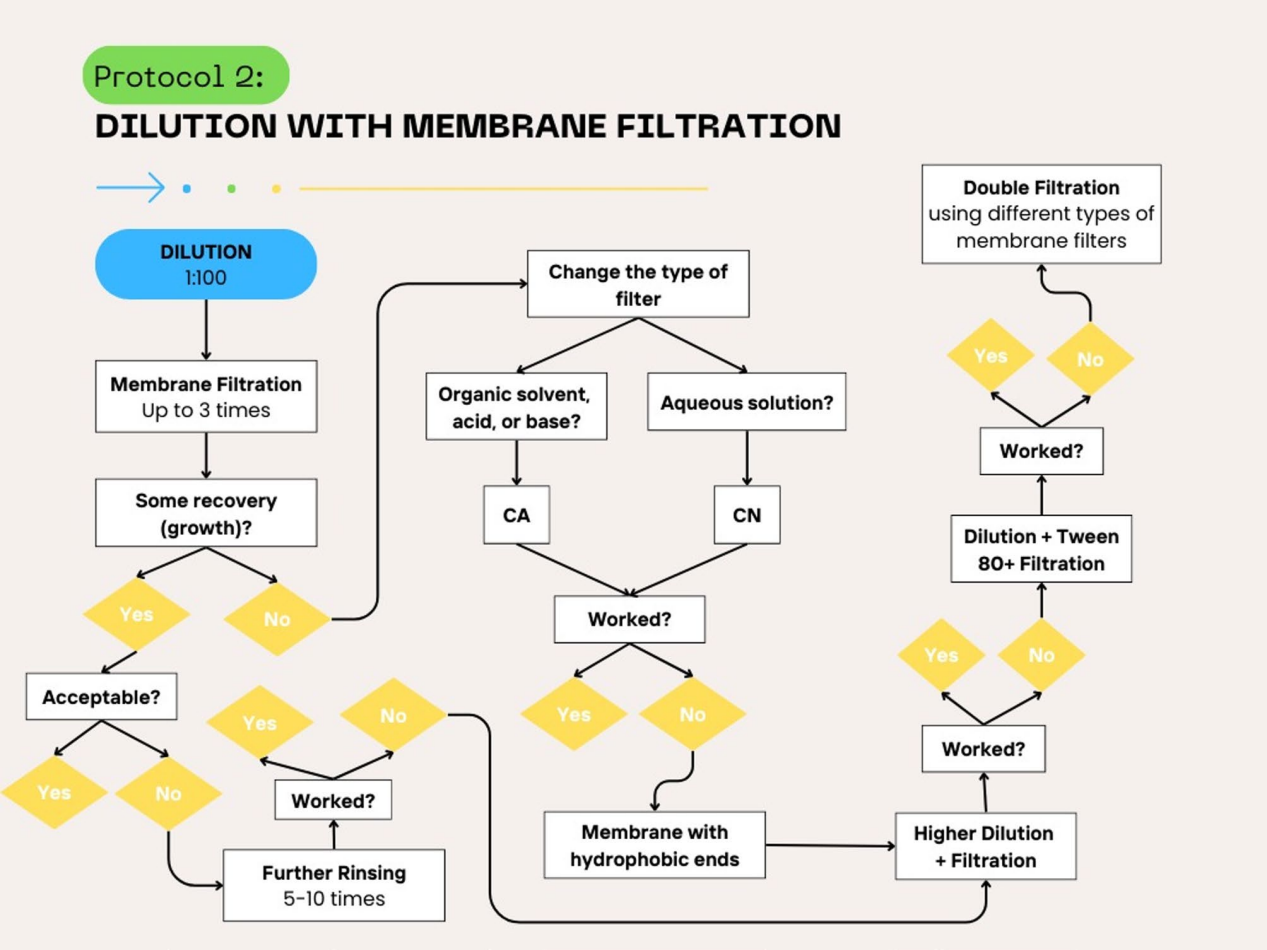
Protocol 2 followed for the optimization of neutralization methods for microbial enumeration of finished products when none of the methods illustrated in Protocol 1 were sufficient for neutralization of antimicrobial activity and further dilution (≥ 1:100) was needed

**Fig. 3:**
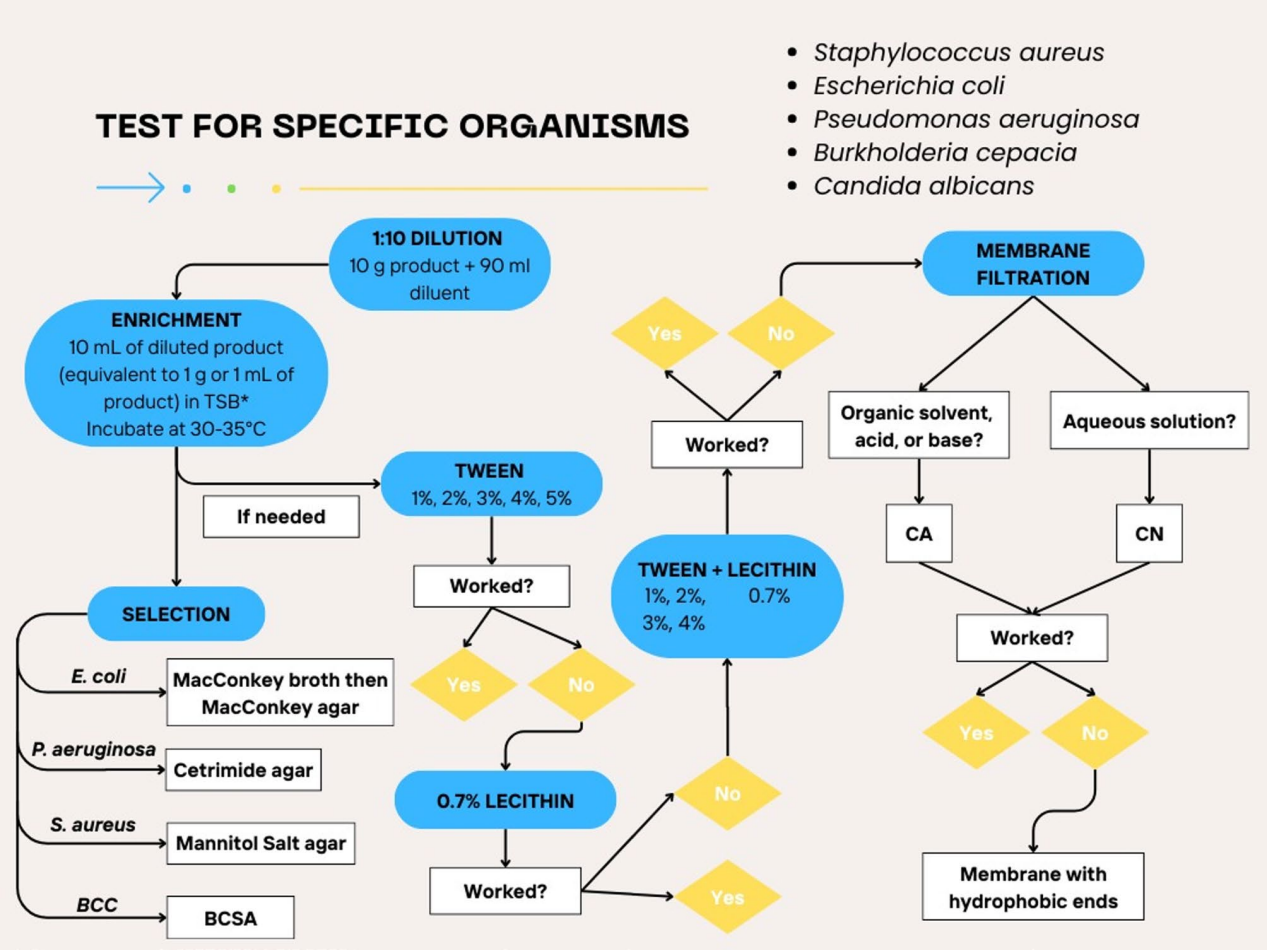
Protocol followed for testing for specific organisms in finished products and optimization of methods for neutralization of antimicrobial activity in the finished products

The procedure followed for method suitability testing is outlined in Figs. 1, 2 and 3. Steps described were followed in the sequence shown until the method tested was found to sufficiently neutralize the preservative or other ingredient(s) that inhibited microbial or fungal growth. This was evident by observing the growth and/or count of standard microbial strains (Table 1) added to the finished product after attempted neutralization and incubation as specified by the USP for each test.

### Determination of total aerobic microbial count

For TAMC (Fig. 1), each product was first diluted 1:10 with pH adjustment to pH 6–8 if necessary. If this was not enough for neutralization (if microbial recovery was not within the acceptable range of 50–200% specified by the USP), 1% tween 80 (with 1% increments up to a final concentration of 4%), 0.7% lecithin, or both were added to the diluent. If none of these were sufficient for neutralization, further dilution of 1:20, 1:30, or 1:50 was done (Fig. 1). For solid dosage forms, 1:100 dilution was tested if smaller dilutions did not work and 0.7% lecithin alone or in combination with 1–4% tween 80 was added if necessary. After the attempted neutralization, TAMC was determined by the pour plate technique. Briefly, 1mL of the sample prepared was added to a 9-cm petri dish with 15–20 ml of molten SCDA. The plates were then incubated at 30–35 °C for 3 to 5 days. SCDA was used for *Staphylococcus aureus*, *Pseudomonas aeruginosa*, *Bacillus subtilis*, *Candida albicans* and *Aspergillus brasiliensis*. The percent recovery was calculated by dividing the mean count of any of the test organisms in the presence of the test product by the value of the control (no product). For both aqueous and solid dosage forms, if none of these methods worked, Protocol 2 involving further dilution (1:100) with membrane filtration was followed (Fig. 2).

### Determination of total yeast and mold count

For TYMC (Fig. 1), each product was first diluted at 1:10 with pH adjustment if necessary. If this method did not work, 1% tween 80 was added to the diluent with 1% increments up to a final concentration of 4%. If none of these methods were sufficient for neutralization, and the product was a solid dosage form, 1:100 dilution was tested and 0.7% lecithin alone or in combination with 1–4% tween 80 was added if necessary. After the attempted neutralization, TYMC was determined by the pour plate technique. Briefly, 1 ml of the sample prepared was added to a 9-cm petri dish with 15–20 ml of molten SDA. The plates were then incubated at 20–25 °C for 5 to 7days for determining the TYMC using *Candida albicans* and *Aspergillus brasiliensis*. The percent recovery was calculated by dividing the mean count of any of the test organisms in the presence of the test product by the value of the control (no product). If the product was an aqueous dosage form or if none of the above methods worked, Protocol 2 (Fig. 2) was followed.

### Further dilution with membrane filtration (Protocol 2)

Finished products for which the aforementioned methods did not work were further diluted, which was followed by membrane filtration (Fig. 2). Suitable amount of the product (representing 1 g or 1 ml of the product) was first diluted at 1:100, membrane filtered up to 3 times (with a membrane filter having a nominal pore size not greater than 0.45 μm). A different membrane filter was used for each organism. The membrane filter was then transferred to the surface of a SCDA or SDA plate. SCDA plates were incubated at 30–35 °C for not less than 3 days and the SDA plates at 20–25 °C for not less than 5 days. SCDA was used for *Staphylococcus aureus*,* Pseudomonas aeruginosa*,* Bacillus subtilis*,* Candida albicans* and *Aspergillus brasiliensis* to determine the TAMC while SDA was used for *Candida albicans* and *Aspergillus brasiliensis* to determine the TYMC. The arithmetic mean of the counts per medium was taken, and the number of CFU in the original inoculum was calculated and compared with the control (no product).

In case of microbial recovery that was not within the acceptable range specified by the USP (50–200%), further rinsing was done 3–5 times to improve the recovery. In case of no microbial recovery, or failure of further rinsing to improve it, then further dilution followed by membrane filtration were done. If this did not work, the same step was repeated with the addition of 1–5.5.5% tween 80. If this was still not sufficient, then double membrane filtration was done, first using a 8 μm filter that allowed adsorption and removal of the antimicrobial to be neutralized, then using a 0.45 μm filter to filter bacteria and/or yeast and molds for growth on culture media. If double filtration did not work, then the type of filter was changed to cellulose acetate (CA) in case of organic solvents, acids, or bases or to cellulose nitrate (CN) in case of aqueous solutions. If these did not work, then a hydrophobic edge membrane was used for filtration.

### Testing for specific organisms

Testing for the absence of specific organisms in specific pharmaceutical dosage forms was done according to USP 62 and to the criteria detailed in the USP 1111 (Table 1). Organisms tested in this study included *S. aureus*, *E. coli*, *P. aeruginosa*, and *B. cepacia* (Fig. 3). Briefly, 1:10 dilution was performed by adding 10 g of the finished product to 90 mL of enrichment medium. For bacterial pathogens, 10 mL of this diluted product (equivalent to 1 g or 1 mL of the finished product) was then used to inoculate soybean-casein digest broth, which was followed by mixing and incubation at 30–35 °C for 18–24 h for *E. coli*, *P. aeruginosa*, and *S. aureus*, and for 48–72 h for *B. cepacia.* If this dilution step was not enough for neutralization, 1% tween 80 was added to the finished product with 1% increments up to a final concentration of 5% and microbial recovery was checked. If this was not sufficient for neutralization, 0.7% lecithin was added. If this did not work, both 0.7% lecithin and 1% up to 4% tween 80 were added. If none of these methods worked, then dilution followed by membrane filtration was done using a CA filter in case of organic solvents, acids, or bases or a CN filter in case of aqueous solutions. If these did not work, then a hydrophobic edge membrane was used for filtration.

Following neutralization, testing for specific organisms was done according to the USP guidelines for each pathogen [5]. The aforementioned enrichment and neutralization steps were followed by a selection step depending on the pathogen investigated. For *E. coli*, the container was shaken, 1 ml of soybean-casein digest broth was transferred to 100 mL of MacConkey broth and incubated at 42–44 °C for 24–48 h. This was then subcultured on a MacConkey agar plate and incubated at 30–35 °C for 18–72 h. Growth of colonies indicated the possible presence of *E. coli*.

For *P. aeruginosa*, the enrichment step was followed by subculturing on a cetrimide agar plate and incubation at 30–35 °C for 18–72 h. Growth of colonies indicated the possible presence of *P. aeruginosa*. This was confirmed by an Oxidase test according to the manufacturer’s instructions. For *S. aureus*, the enrichment step was followed by subculturing on a Mannitol salt agar plate and incubation at 30–35 °C for 18–72 h. The possible presence of *S. aureus* was indicated by the growth of yellow or white colonies surrounded by a yellow zone, which was confirmed by identification tests. These included subculturing on a SCDA plate, incubation at 30–35 °C for 18–72 h and observing for golden colonies which indicate a positive result. Another identification test used was the Staphylase kit (Oxoid) in which a positive result was indicated by the agglutination of the blue test latex particles within 20 s.

For *B. cepacia* complex, the enrichment step was followed by subculturing on a *B. cepacia* selective agar (BCSA) plate and incubation at 30–35 °C for 48–72 h. The possible presence of *B. cepacia* was indicated by the growth of greenish-brown colonies with yellow halos, or white colonies surrounded by a pink-red zone on BCSA. Any growth on BCSA was confirmed by identification tests.

For *Candida albicans*, the enrichment step was done by inoculating a suitable amount of Sabouraud dextrose broth with a quantity corresponding to 1 g or 1 ml of the product, mixing, and incubating at 30° to 35° for 3 to 5 days. This was followed by a selection step done by subculturing on a SDA plate and incubating at 30° to 35° for 24 to 48 h. Growth of white colonies indicated the possible presence of *C. albicans*, which was confirmed by identification tests.

## Results

### Pharmaceutical finished products under investigation

Method suitability testing was done for 133 pharmaceutical finished products. Of these, 40 finished products required optimization of the neutralization method through multiple steps of testing. For 18 of these 40 finished products, neutralization was achieved through 1:10 dilution with warming of the diluent at 45 °C (to ensure proper emulsification of water-insoluble substances). For the remaining 22 products, further optimization was required as described below. These 22 finished products, their product codes, pharmaceutical dosage forms, active ingredients and their concentrations, and inactive ingredients are described in Table 2.

**Table 2:** List of finished products tested in this study, their active pharmaceutical ingredients, concentrations, and inactive ingredients

	Product code	Dosage form	APIs	Concentrations	Inactive ingredients
1	BOA101	Oral Solution	Aripiprazole	5 mg	Sucrose, Glycerol, liquid sorbitol (non-crystallizing), monopropylene glycol, lactic acid, poloxomer 407, methylparaben, disodium edetate, propylparaben, sodium hydroxide, purified water
2	BOA102	Oral Suspension	Nifuroxazide	220 mg	Avicel CL 611, Carboxymethyl cellulose sodium Cremophor RH 40, Glycerol, Sucrose, Methylparaben, Citric acid monohydrate, Banana flavor, Purified water
3	BOA103	Oral Solution	Atomoxetine hydrochloride	22.856 mg (equivalent to 20 mg Atomoxetine)	Anhydrous sodium dihydrogen phosphate, Sodium benzoate, Liquid sorbitol (non-crystallizing), Xylitol, Sucralose, Strawberry flavor BC 501 (Ethyl Acetate, Ethyl maltol, Ethyl Butyrate, Amyl Acetate, Vanillin, Cis-3-Hexenol, Monopropylene glycol), Banana flavor BC 301 (lsoamyl Isovalerate, Isoamyl Acetate, Vanillin, Ethyl Acetate, Butyric acid, Monopropylene Glycol), Phosphoric acid, Sodium hydroxide, Purified water
4	BTA101	Cosmetic lotion	N/A		96% ethyl alcohol, Lepidium meyenii root extract, Procapil‡, Glycerin, D-Panthenol, Vanillylbutyl ether, Anhydrous caffeine, Rosmarinus oficinalis leaf extract, Creasoluble N1₶, Ucare Polymer JR-400₽, Cetrimonium bromide, Esculin, Sodium shale oil sulfonate, Lavender oil
5	BTA102	Medicated Shampoo	Ketoconazole	2 g	Methylparaben, Propylparaben, EDTA disodium, Polyquaternium-10, Sodium laureth sulphate 27%, Butylated hydroxytoluene, Glycerol, Cocamidopropyl betaine 30%, Sodium chloride, Anhydrous Citric Acid, Ponceau 4R, Purified water
6	BTA103	Cosmetic Shampoo	N/A	N/A	Sodium lauryl sulfate, Cocamidopropyl glutamate, Sodium cocoyl glutamate, Polyacrylate crosspolymer-6, Propylene glycol, Euperlan PK 400₹, Crambe abyssinica seed oil, PEG-7 glyceryl cocoate, Abil ME 45₺, Diacaprilyl ether, Lauryl alcohol, Tocopherol, Varisoft Eq. 100₫, Viatenza^®^ Mongongo PO6 Amphiphilic₾, Sodium chloride, Inebact, Creasoluble N1₶, Hydra restructuring 1,604,802, Unicert yellow 08005-J, Unicert red 07004-J
7	BTA104	Topical solution	Luliconazole	10 mg	Polyethylene glycol 400, Medium chain triglycerides, Phosphoric acid, Isopropyl alcohol, Anhydrous ethanol
8	BTA105	Topical ointment	Mometasone furoate Salicylic acid	1 mg 50 mg	Beeswax, Liquid paraffin, White petrolatum
9	BTA106	Topical ointment	Calcipotriol monohydrate	52.2 mcg	dl-α-tocopherol, Ascorbyl tetraisopalmitate, Light liquid paraffin, White petrolatum, PPG-15 stearyl ether
10	BTA107	Topical cream	Polymyxin B Sulphate Neomycin Pramoxine HCl	10,000 U 3.5 mg 10 mg	Sodium metabisulphite, Cetostearyl alcohol, Polawax GP 200, Miglyol 812, Cremophore A6, Cremophore A25, Glyceryl monostearate, Softisan 378, Propylene glycol, Methylparaben, Propylparaben, Sodium hydroxide, Purified water
11	BTA108	Topical gel	Diphenhydramine Hydrochloride Zinc Acetate Menthol	20 mg 1 mg 10 mg	Ethyl alcohol 96%, Hydroxypropyl Cellulose (Klucel MF), Purified water
12	BOS101	Hard gelatin capsules	Sodium bicarbonate	500 mg	Sucrose, Colloidal silicon dioxide (Aerosil 200), Magnesium stearate, Gelatin, Methylparaben, Propylparaben, Sodium lauryl sulfate, Aerosil, Titanium dioxide [C.I. No. 778911], Quinoline yellow [CI No. 47005], Erythrosine red [CI No. 45430]
13	BOS102	Hard gelatin capsules	Ferric hydroxide polymaltose Folic acid Cyanocobalamin	101.69 mg (Equivalent to 30 mg elemental iron) 0.5 mg 0.0025 mg	Dicalcium phosphate dihydrate, Microcrystalline cellulose PH 101, Sodium starch glycolate, Hydroxypropyl cellulose (LF,75–150 cps), Sodium lauryl sulphate, Sodium stearyl fumarate, Colloidal silicon dioxide (Aerosil 200), Purified water Gelatin, Methylparaben, Propylparaben, Aerosil, Titanium dioxide: CIN 77,891, Sunset yellow: CIN 15,985, Carmoisine red: CIN 14,720, Brilliant blue CIN 42,090, Yellow Iron Oxide
14	BOS103	Film-coated tablets	Clopidogrel bisulfate	97.875 (equivalent to clopidogrel 75 mg)	Lactose monohydrate, Hydroxypropylcellulose, Crosmcarmellose sodium, Sodium stearyl fumarate, Advantia™ prime
15	BOS104	Hard gelatin capsules	Nifuroxazide	200 mg	Maize starch, Lactose monohydrate, Pregelatinized starch, Magnesium stearate, Colloidal Silicon dioxide, Gelatin, Titanium dioxide: CIN 77,891, Sunset yellow: CIN 15,985, Brilliant blue CIN 42,090, Quinoline yellow: CIN 47,005
16	BOS105	Hard gelatin capsules	Nifuroxazide	100 mg	Maize starch, Lactose monohydrate, Pregelatinized starch, Magnesium stearate, Colloidal Silicon dioxide (Aerosil 200), Gelatin, Methylparaben, Propylparaben, Sodium lauryl sulfate, Titanium dioxide: CIN 77,891, Quinoline yellow: CIN 47,005, Carmoisine red: CIN 14,720
17	BOS106	Hard gelatin capsules	Erdosteine	300 mg	Lactose monohydrate, Croscarmellose sodium, Povidone K25, Magnesium stearate, Silicon dioxide, Gelatin, Titanium dioxide, Ferric oxide yellow
18	BOS107	Film-coated tablets	Eperisone hydrochloride	100 mg	Lactose monohydrate (200 mesh), Croscarmellose sodium, Hydroxypropyl cellulose (LF,75–150 cps), Maize starch, Microcrystalline cellulose PH 101, Magnesium stearate, Aquarius prime BAP 319,750 (Purple)*
19	BOS108	Film-coated tablets	Eperisone hydrochloride	50 mg	Lactose monohydrate (200 mesh), Croscarmellose sodium, Hydroxypropyl cellulose (LF,75–150 cps), Maize starch, Microcrystalline cellulose PH 101, Magnesium stearate, Aquarius prime BAP 319,750 (Purple)*
20	BOS109	Tablets	Azithromycin dihydrate	628.80 mg (equivalent to 600 mg anhydrous Azithromycin)	Calcium phosphate dibasic dihydrate, Croscarmellose sodium, Opadry tm white (07F28588)**, Pregelatinized starch, Poloxamer 407, Magnesium stearate, Aerosil 200, Saccharin sodium
21	BOS110	Tablets	Azithromycin dihydrate	262 mg (equivalent to 250 mg Azithromycin)	Calcium phosphate dibasic dihydrate, Croscarmellose sodium, Opadry tm white (07F28588)**, Pregelatinized starch, Poloxamer 407, Magnesium stearate, Aerosil 200, Saccharin sodium
22	BOS111	Film-coated tablets	Levofloxacin hemihydrate	512.45 mg (equivalent to 500 mg anhydrous Levofloxacin)	Microcrystalline cellulose (Vivapur PH 101), Croscarmellose sodium, Povidone K25, Magnesium stearate, Colloidal silicon dioxide, Advantia™ prime 371915BA01*

*Hydroxypropyl methycellulose 400 cP, Polyethylene, Glycol 400, Titanium dioxide (C1N:7789I), Ponceau 4R, Red Iron Oxide, FD&C Blue No.1 Aluminum Lake

**Hypromellose 15 mPas, Talc, Titanium dioxide, Hypromellose 3 mPas, Hypromellose 50 mPas, Hypromellose 6 mPas, Macrogol 3350, Saccharin sodium

^‡^Water, Butylene glycol, PPG-26-buteth-26, PEG-40 hydrogenated castor oil, Apigenin, Oleanolic acid, Biotinoyl tripeptide-1

^₶^PPG-26-buteth-26, PEG-40 hydrogenated castor oil, water

^₽^Polyquaternium 10, Sodium acetate, Sodium chloride, lsopropyl alcohol, water

^₹^Water, Glycol Distearate, Laureth-4, Cocamidopropyl Betaine, Citric acid, Glycerin, Lactic Acid

^₺^Silicone Quaternium-22, Polyglyccry1-3 Caprate, Dipropylene Glycol, Cocamidopropyl Betaine, Palmitamidopropyltrimonium Chloride, Propylene glycol, water

^₫^Bis-(lsostearoyl/Oleoyl Isopropyl) Dimonium Methosulfate

^₾^*Schinziophyton rautanenii* Kernel Oil Polyglyceryl-6 Esters

### Evaluation of the neutralization procedure toxicity to standard strains

Potential toxicity of neutralizing agents on standard microbial strains was tested. There was an acceptable microbial recovery of at least 84% for all standard strains with all neutralization methods as shown in Table 3. The percent recovery ranged from 84% to 105%, indicating minimal to no effect on growth of the standard strains. Negative control samples showed no microbial growth. For the fungal strains, *Candida albicans* and *Aspergillus brasiliensis*, there was some variation of percent recovery (although not significant) when grown on SCDA (TSA) or SDA.

**Table 3:** Evaluation of the neutralization procedure toxicity to standard strains

Neutralization method	*Staphylococcus aureus*	*Bacillus subtilis*	*Pseudomonas aeruginosa*	*Candida albicans*		*Aspergillus brasiliensis*	
				TSA	SDA	TSA	SDA
1% Tween 80	90%	98%	97%	94%	92%	91%	84%
3.5% Tween 80	93%	97%	90%	95%	93%	93%	94%
1% Tween 80 + 0.7% Lecithin	102%	102%	102%	105%	101%	102%	104%
5.5% Tween 80 + 0.7% Lecithin	98%	99%	99%	98%	99%	98%	98%

*TSA* Tryptic soy agar, *SDA* Sabouraud dextrose agar

### Method suitability testing and selection of the optimal neutralization method

**Fig. 4:**
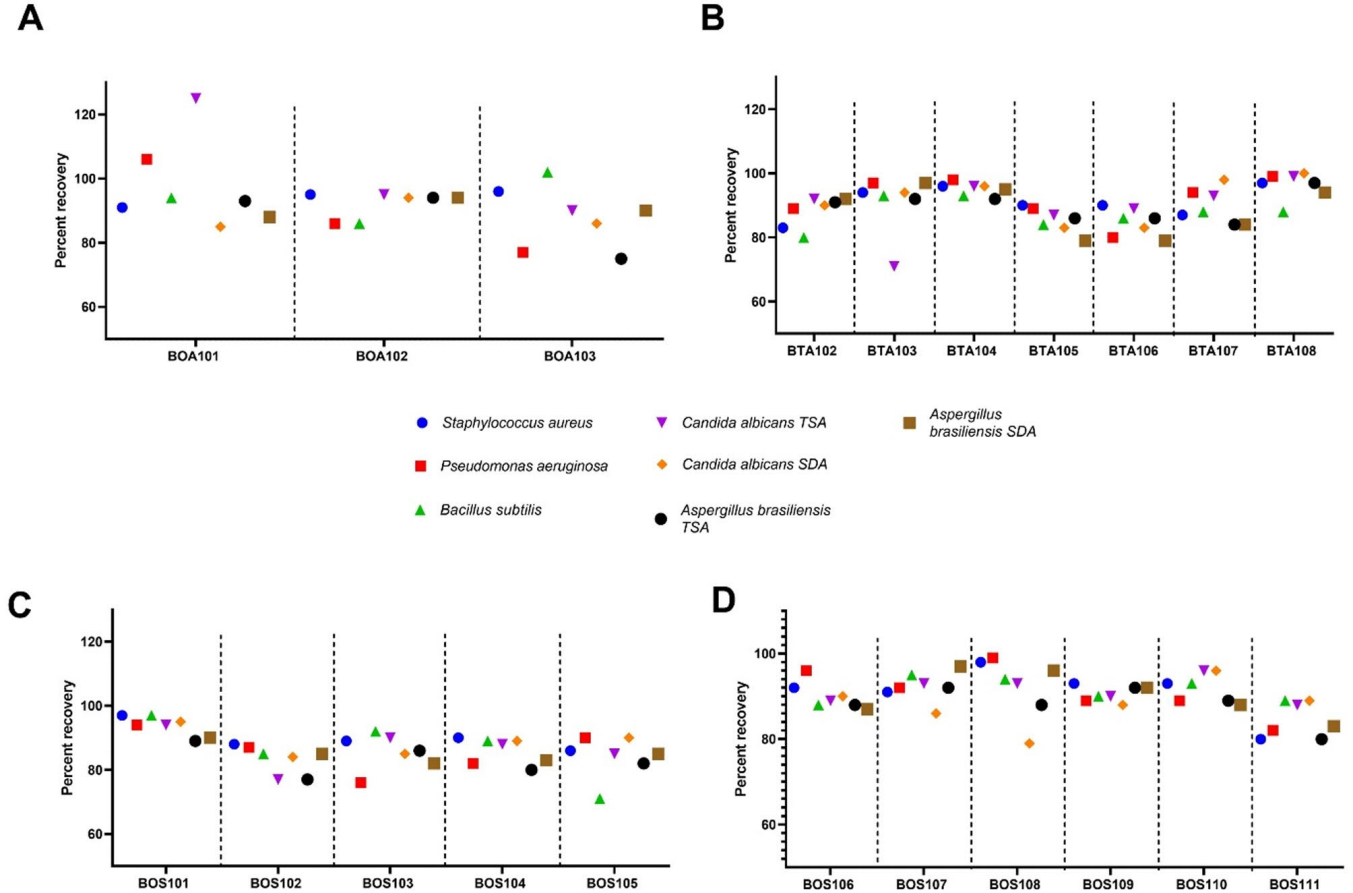
Percent microbial recovery of different standard organisms after using the optimized neutralization procedure for each pharmaceutical finished product. **A** Aqueous oral dosage forms, (**B**) Topical aqueous products, **C** and **D**. Oral solid dosage forms. Shown are arithmetic means of recovery percentages compared with control (no product). TSA, tryptic soy agar; SDA, Sabouraud dextrose agar

The 22 finished products that required further optimization of neutralization methods, the ingredients with antimicrobial activity that they contained along with the neutralization method eventually selected are shown in Tables 4 and 5. Microbial recovery for each product compared to control is shown in Fig. 4 (for each tested bacteria or fungi). Of the finished products tested, there were two oral solutions, one containing parabens while the other sodium benzoate as preservatives, and there was one topical solution preserved with parabens. The use of 1:10 dilution + 1% tween 80 achieved neutralization for both oral solutions (BOA101 and BOA103) and for the TAMC of the topical solution (BTA104). For the TYMC of the topical solution, neutralization was achieved through 1:400 dilution with 1% tween 80 followed by filtration using a CN hydrophobic edge membrane. There were two hair shampoos among the finished products tested. One of these (BTL102) had an active ingredient with inherent antimicrobial activity (ketoconazole) as well as polyquaternium-10 with preservative activity. Neutralization was achieved through 1:100 dilution followed by filtration using a CN or CA membrane filter with three rinses. The other shampoo (BTA103) was a cosmetic product with no API and dilution + 1% tween 80 were enough for neutralization.

**Table 4:** Preservatives contained in, and inherent antimicrobial activity of finished products with challenging method suitability testing

Product	API with inherent antimicrobial activity	Preservative or inactive ingredient with antimicrobial activity	Method that achieved recovery within the acceptable USP limits)
BOA101	None	Parabens	1:10 Dilution (diluent warmed at 45 °C) + 1% tween 80
BOA102	Nifuroxazide	Parabens	**TAMC**: 1:100 + 5.5% tween 80 + 0.7% lecithin **TYMC**: 1:10 Dilution with manual shaking
BOA103	None	Sodium benzoate	1:10 Dilution + 1% Tween
BTA101	N/A	96% ethyl alcohol, cetrimonium bromide	1:100 dilution + single membrane filtration (CN) wo HE, (0.45 μm) **TAMC and TYMC**: 1:100 dilution + membrane filtration
BTA102	Ketoconazole	Parabens, Polyquaternium-10	1:100 dilution + filtration using a CN or CA membrane with 3 rinses
BTA103	N/A	Sodium lauryl sulfate, Tocopherol (mild antibacterial activity)	1:10 Dilution + 1% Tween 80 **TAMC and TYMC**: 1:10 Dilution + enrichment
BTA104	Luliconazole	Isopropyl alcohol	**TAMC**: 1:10 Dilution + 1% tween 80 **TYMC**: 1:400 dilution, 1% tween 80 + filtration using a hydrophobic edge CN membrane
BTA105	Salicylic acid	N/A	1:10 Dilution (diluent warmed at 45 °C) + 1% tween 80
BTA106	N/A	dl-α-tocopherol, Ascorbyl tetraisopalmitate (mild antibacterial activity)	1:10 Dilution (diluent warmed at 45 °C) + 1% tween 80
BTA107	Polymyxin B sulphate and Neomycin	Parabens	1:200 dilution + double filtration with 5 rinses using an 8µM MCE prefilter then a 0.45µM CN filter
BTA108	Menthol	Ethyl alcohol	1:10 Dilution + 1% tween 80
BOS101	N/A	Parabens	1:50 Dilution (diluent warmed at 45 °C) + 3.5% tween 80
BOS102	N/A	Parabens	1:10 Dilution (diluent warmed at 45 °C) + 1% tween + 0.7% lecithin
BOS103	N/A	N/A	1:10 Dilution + pH adjustment
BOS104	Nifuroxazide	N/A	**TAMC**: 1:100 Dilution (diluent warmed at 45 °C) + double filtration with 3 rinses using an 8µM MCE prefilter then a 0.45µM CN filter **TYMC**: 1:10 Dilution (diluent warmed at 45 °C)
BOS105	Nifuroxazide	N/A	**TAMC**: 1:100 Dilution (diluent warmed at 45 °C) + double filtration with 3 rinses using an 8µM MCE prefilter then a 0.45µM CN filter **TYMC**: 1:10 Dilution (diluent warmed at 45 °C)
BOS106	N/A	N/A	1:10 Dilution (diluent warmed at 45 °C) + pH adjustment
BOS107	N/A	N/A	1:100 dilution + double filtration with 3 rinses using an 8µM MCE prefilter then a 0.45 µM CA or CN filter
BOS108	N/A	N/A	1:100 dilution + double filtration with 3 rinses using an 8µM MCE prefilter then a 0.45 µM CA or CN filter
BOS109	Azithromycin	N/A	**TAMC**: 1:200 Dilution + double filtration with 5 rinses using an 8 μm MCA prefilter then a CA filter **TYMC**: 1:10 Dilution
BOS110	Azithromycin	N/A	**TAMC**: 1:200 Dilution + double filtration with 5 rinses using an 8 μm MCA prefilter then a CA filter **TYMC**: 1:10 dilution
BOS111	Levofloxacin	N/A	1:100 Dilution + double filtration with 3 rinses using an 8µM MCE prefilter then a 0.45µM CN filter **TYMC**: 1:10 Dilution

*MCE* Mixed cellulose ester, *CA* Cellulose acetate, *CN* Cellulose nitrate

**Table 5:** Selected method of neutralization for each finished pharmaceutical product based on method suitability testing

	Dosage form	Product	Microbial enumeration	Test for specific pathogens
1	Oral solution	BOA101	1:10 Dilution (diluent warmed at 45 °C) + 1% tween 80	1:10 Dilution
2	Suspension	BOA102	**TAMC**: 1:100 + 5.5% tween 80 + 0.7% lecithin **TYMC**: 1:10 Dilution with manual shaking	1:10 Dilution
3	Oral solution	BOA103	1:10 Dilution + 1% tween 80	1:10 Dilution
4	Cosmetic lotion	BTA101	1:100 Dilution + filtration using a 0.45µM CN membrane	1:100 Dilution + membrane filtration
5	Shampoo	BTA102	1:100 Dilution + filtration using a CA or CN membrane with 3 rinses	1:100 Dilution + filtration using a CN or CA membrane with 3 rinses
6	Cosmetic shampoo	BTA103	1:10 Dilution + 1% tween 80	1:10 Dilution
7	Topical solution	BTA104	**TAMC**: 1:10 + 1% tween 80 **TYMC**: 1:400 Dilution + 1% tween 80 + filtration using a hydrophobic edge CN membrane	1:10 Dilution + 1% tween 80
8	Topical ointment	BTA105	1:10 Dilution (diluent warmed at 45 °C) + 1% tween 80	1:10 Dilution
9	Topical ointment	BTA106	1:10 Dilution (diluent warmed at 45 °C) + 1% tween 80	1:10 Dilution
10	Topical Cream	BTA107	1:200 Dilution + filtration using an 8µM MCE prefilter then a 0.45µM CA filter with 5 rinses	1:200 Dilution + filtration using an 8µM MCE prefilter then a 0.45µM CA filter with 5 rinses
11	Topical gel	BTA108	1:10 Dilution + 1% tween 80	1:10 Dilution
12	Capsules	BOS101	1:50 Dilution (diluent warmed at 45 °C) + 3.5% tween 80	1:10 Dilution
13	Capsules	BOS102	1:10 Dilution (diluent warmed at 45 °C) + 1% tween 80 + 0.7% lecithin	1:10 Dilution
14	Tablets	BOS103	1:10 Dilution + pH adjustment	1:10 Dilution
15	Capsules	BOS104	**TAMC**: 1:100 Dilution (diluent warmed at 45 °C) + filtration using an 8µM MCE prefilter then a 0.45µM CN filter with 3 rinses, **TYMC**: 1:10 Dilution (diluent warmed at 45 °C)	1:10 Dilution (diluent warmed at 45 °C) + double filtration using an 8µM MCE prefilter then a 0.45µM CN filter with 3 rinses
16	Capsules	BOS105	**TAMC**: 1:100 Dilution (diluent warmed at 45 °C) + filtration using an 8µM MCE prefilter then a 0.45µM CN filter with 3 rinses, **TYMC**: 1:10 Dilution (diluent warmed at 45 °C)	1:10 Dilution (diluent warmed at 45 °C) + filtration using an 8µM MCE prefilter then a 0.45µM CN filter with 3 rinses
17	Capsules	BOS106	1:10 Dilution (diluent warmed at 45 °C) + pH adjustment	1:10 Dilution
18	Tablets	BOS107	1:100 Dilution + filtration using an 8µM MCE prefilter then a 0.45µM CA or CN filter with 3 rinses	1:100 Dilution + double filtration using an 8µM MCE prefilter then a 0.45µM CA or CN filter with 3 rinses
19	Tablets	BOS108	1:100 Dilution + filtration using an 8µM MCE prefilter then a 0.45µM CA or CN filter with 3 rinses	1:100 Dilution + double filtration using an 8µM MCE prefilter then a 0.45µM CA or CN filter with 3 rinses
20	Tablets	BOS109	**TAMC**: 1:200 Dilution + filtration using an 8µM MCE prefilter then a 0.45µM CA with 5 rinses **TYMC**: 1:10 Dilution	1:200 Dilution + double filtration using an 8µM MCE prefilter then a 0.45µM CA filter with 5 rinses
21	Tablets	BOS110	**TAMC**: 1:200 Dilution + filtration using an 8µM MCE prefilter then a 0.45µM CA with 5 rinses **TYMC**: 1:10 Dilution	1:200 Dilution + double filtration using an 8µM MCE prefilter then a 0.45µM CA filter with 5 rinses
22	Tablets	BOS111	**TAMC**: 1:200 Dilution + filtration using an 8µM MCE prefilter then a 0.45µM CN with 3 rinses **TYMC**: 1:10 Dilution	1:100 Dilution + double filtration using an 8µM MCE prefilter then a 0.45µM CN filter with 3 rinses

There was one oral suspension (BOL102) with parabens as a preservative and inherent antimicrobial activity of its active ingredient (nifuroxazide). Neutralization was successful through 1:100 dilution + 5.5% tween 80 + 0.7% lecithin for total aerobic microbial count and 1:10 dilution with manual shaking for total yeast and mold count. There were two ointments (BTA105 and BTA106), one of which (BTA105) contained salicylic acid as an API. For both ointments, neutralization was achieved through 1:10 dilution (with diluent warmed at 45 °C) + 1% tween 80. The only cream dosage form was a topical antibiotic cream (BTA107) containing Polymyxin B Sulphate and neomycin as APIs and parabens as preservatives. Neutralization required 1:200 dilution followed by double filtration with 5 rinses using an 8 µM mixed cellulose ester (MCE) prefilter followed by a 0.45 µM CA filter. There was one topical gel (BTS104) that contained menthol as an API and 96% ethyl alcohol, both with antimicrobial activity. Dilution (1:10) with 1% tween 80 achieved neutralization for this gel (Table 4).

For solid dosage forms tested, 5 finished products were capsules and 7 were tablets. Two of the capsule dosage forms (BOS101 and BOS102) had parabens as preservatives and no inherent antimicrobial activity to their APIs. Neutralization was achieved through 1:50 dilution (diluent warmed at 45 °C) with the addition of 3.5% tween 80 for BOS101 and 1:10 dilution (diluent warmed at 45 °C) with the addition of 1% tween 80 and 0.7% lecithin for BOS102. Another two of the capsule finished products (BOS104 and BOS105) contained different concentrations (100 mg and 200 mg) of the API nifuroxazide with inherent antimicrobial activity. For both products, neutralization was achieved using 1:100 Dilution (diluent warmed at 45 °C) followed by double filtration with 3 rinses using an 8 µM MCE prefilter then a 0.45 µM CN filter for TAMC. For TYMC, neutralization required 1:10 dilution with the diluent warmed at 45 °C. The last capsule finished product (BOS106) had no inherent antimicrobial activity to its API (erdosteine) and was neutralized through 1:10 dilution with pH adjustment (Tables 4 and 5). That indicates that pH is an important factor that needs to be considered for proper microbial recovery.

Four of the 7 tablet finished products contained an antibiotic as an API. BOS109 and BOS110 contained 250 and 600 mg of azithromycin, respectively. For both products, neutralization was achieved through 1:10 dilution for TYMC and through 1:200 Dilution followed by double filtration with 5 rinses using an 8µM MCA prefilter, then a CA filter for TAMC. BOS111 contained 500 mg of levofloxacin. It was neutralized through a 1:10 dilution for TYMC and through 1:200 dilution followed by double filtration using an 8µM MCE prefilter then a 0.45µM CN with 3 rinses for TAMC. Another tablet dosage form (BOS103) had no inherent antimicrobial activity to its API (clopidogrel) and was neutralized through 1:10 dilution with pH adjustment. The remaining two tablet dosage forms (BOS107 and BOS108) contained two different concentrations (50 mg and 100 mg, respectively) of the active ingredient eperisone hydrochloride. Neutralization in both products was achieved through 1:100 dilution followed by double filtration with three rinses using an 8 µM MCE prefilter then a 0.45 µM CA or CN filter (Tables 4 and 5) (Fig. 4).

### Testing for specific microorganisms

Testing for the absence of specific organisms was done according to the pharmaceutical dosage form as described in the USP (Table 1) and method suitability was optimized following the protocol outlined in Fig. 3. For 11 of the 22 finished products that required multiple steps of optimization, neutralization was achieved for testing for specific microorganisms using the same neutralization method that worked for microbial enumeration (Table 5). These products were BTA104 (1:10 dilution + 1% tween 80); BTL101, BTL102 (1:100 dilution + filtration with 0.45 μm CN membrane); BTA107, BOS104, BOS105, BOS107, BOS108, and BOS111 (1:100 dilution + double filtration with 3 rinses using an 8 μm MCE prefilter then a 0.45 μm CA or CN filter); and BOS109, BOS110 (1:200 dilution + double filtration with 5 rinses using an 8 μm MCE prefilter then a 0.45 μm CA filter).

Another 8 of these 22 products were neutralized using 1:10 dilution with enrichment on selective media, a step specified in the USP only for testing for specific organisms (detailed in the methods section). These products were BOA101, BOA103, BTA103, BTA105, BOS103, and BOS106. Neutralization for microbial enumeration of these 6 products was achieved through 1:10 dilution with tween 80 and/or lecithin with no membrane filtration (Table 5). For the remaining 3 products (BOA102, BTA106, and BTS104), 1:10 dilution was enough for neutralization for testing for the absence of specific organisms despite further steps required for neutralization in case of microbial enumeration (Table 5).

## Discussion

Microbial limit tests for non-sterile pharmaceutical finished products verify that the risk of microbial multiplication during use and storage has been properly addressed through different phases of the manufacturing process including the addition of preservatives, and environmental risk control. This is especially important since the individuals using the medication could be more vulnerable to infection or even immunocompromised, potentially leading to infection or even death. As early as 1902, there was a reported incident of a medicament-related infection. Due to contamination of a batch of the plague vaccine with tetanus bacilli, 19 people died in Mulkowal, India following vaccine administration [6, 7]. There have been other reports in the past century [8, 9]. More recently, a study between 2007 and 2008 found that 50% of drugs tested sampled from a hospital outpatient pharmacy in Dar es Salaam, Tanzania were heavily contaminated [10]. Contaminants included *Klebsiella*,* Bacillus*, and *Candida* species and some of the isolates displayed antibiotic resistance [10]. Similar results were reported in a study that collected samples between April and June 2021 [11].

For microbial limit testing and testing for the absence of specified organisms, antimicrobial activity inherent to the API or due to an added preservative or excipient must be sufficiently neutralized to allow the growth and recovery of the added microbial inocula [3, 12]. Neutralization of common preservatives is often successful through dilution of the finished product. When a dilution factor of 1:10 does not neutralize the antimicrobial activity, higher dilution factors are often the next steps tested. In our study, adjustment of the diluted product to a neutral pH (6–8), was enough to allow neutralization without resorting to dilution factors higher than 1:10. Extreme pH values are known to impact method suitability testing [13] and pH adjustment is a minor step that is frequently overlooked and that can help avoid unnecessary additional optimization steps. This adjustment worked for two of our solid dosage forms (BOS103 tablets and BOS106 capsules).

While dilution usually neutralizes many common preservatives, neutralization of some products may prove to be more challenging especially if they contain APIs with antimicrobial properties. In this study, all finished products that contained parabens or sodium benzoate as the only ingredient with antimicrobial activity (BOA101, BOA103, BOS101, and BOS102) were neutralized through 1:10 dilution with or without the addition of tween 80 or lecithin. On the other hand, all antimicrobial products required multiple steps of optimization and could only be neutralized through a membrane filtration step. These contained antibiotic or antifungal APIs such as nifuroxazide, azithromycin, polymyxin B sulfate, neomycin, levofloxacin, ofloxacin, ketoconazole, or luliconazole. It is often assumed that antibiotic and antifungal finished products will likely not contain contaminants beyond the limits allowed by the USP due to their inhibitory effects on microbial growth. However, this is likely not true for narrow spectrum antimicrobials and for antimicrobial-resistant contaminants, which represent an increasing global threat. One study found that all drugs randomly sampled for microbiological QC contained microbial contaminants, including antibiotic solid dosage forms such as amoxicillin and doxycycline [10]. The same study reported that *Bacillus* and *Klebsiella* isolates from some of the drug samples were resistant to Augmentin^®^ and cloxacillin, which are broad spectrum antibiotics often used as first-line treatments [10]. Other factors that can contribute to challenging neutralization of a given pharmaceutical product include extreme pH values, matrix insolubility in aqueous media, and adverse reactions (e.g. heat generation or swelling) with the solution medium [13].

In this study, we mainly tested two chemical neutralizers while optimizing our neutralization protocols, namely tween 80 and lecithin. This was based on recommendations detailed in USP Chap. 61 according to the type of interfering substance or preservative present in the pharmaceutical preparation. Since most preparations in our study contained parabens, we used lecithin and tween 80 as well as dilution as recommended. Tween 80 and lecithin are two widely used neutralizers that are available in most microbiological QC laboratories and were sufficient for neutralization in many cases in our study. It is recommended, however, to test other neutralizers especially when multiple preservatives and/or excipients are present in the formulation. Examples include histidine, sodium thiosulfate, thioglycolate, and glycine. Further work is needed to describe detailed evaluation of a broader neutralization panel, particularly for preparations that are challenging to neutralize.

Membrane filtration following product dilution allows using higher dilution factors for neutralization of challenging antimicrobial ingredients, while allowing concentration of the existing microbes on the membrane surface. Higher dilution factors can have the added benefits of ease of sample handling and processing and ease of enumeration on solid culture media [14]. Different types of filters have been tested in this study. We used cellulose nitrate filters for aqueous solutions and cellulose acetate for organic solvents, acids or bases. With higher dilution factors and lower microbial count, there is a high likelihood of low recovery of some of the contaminants. Hydrophobic edge membranes are useful in these cases. The non-wetting edge acts as a seal which prevents wicking of the liquid filtered under the sealing rim of the filter holder, ensuring containment and accuracy of the filtration process. Filtration with hydrophobic edge membranes was the selected neutralization method for the TYMC of BTA104, which required a large dilution factor (1:400) for neutralization to be successful.

The negative impact of not following up on products that are challenging to neutralize until neutralization is successful in microbiological QC laboratories is often underestimated. The assumption that that the inhibited organism due to potential failure of neutralization will not be present in the finished product, is not in contradiction with the USP specifications [3]. While optimization of neutralization methods can be tedious and time consuming, it is an important step to avoid the risk of contaminants in the final product beyond what is allowed by the USP. The detailed optimization protocol described here can help guide microbiology QC laboratorians to the suitable method for each product, especially ones with similar APIs or excipients.

We established method suitability for testing for the absence of *Burkholderia cepacia* complex (BCC) in all aqueous finished products in our study. BCC is a group of 24 closely related opportunistic pathogenic species that represent a feared contaminant of aqueous pharmaceutical and cosmetic products [15, 16]. They are a frequent reason for recall of non-sterile pharmaceutical products [17, 18] and nosocomial outbreaks especially threatening to immunocompromised individuals [19, 20] and cystic fibrosis patients [21, 22].

In 2019, the USP published a new chapter (Chapter < 60>) detailing methods for testing for BCC in water-based finished products which can also be used for testing water used in the manufacturing process [23, 24]. Testing for BCC in non-sterile aqueous products is mandatory [23] and the FDA recommends that manufacturers follow the procedures described in the compendial test published by the USP [25]. However, BCC contaminants are not frequently reported, especially in developing countries [15]. This may be associated with less rigorous compliance or with difficulty in BCC identification. BCC has been previously reported to be misidentified as *E. coli* or *Pseudomonas* spp [26]. No special requirements were needed for the recovery of BCC compared to the standard aerobic method. This is in fact an important conclusion, as it demonstrates that the procedures described are sufficient for BCC detection. The protocols described here can help guide QC laboratories seeking to include routine testing for BCC in aqueous products and raw materials.

One limitation of our study is the potential failure of all combinations of the neutralization measures tested here to neutralize the antimicrobial activity in a given preparation. While there were no such incidents with any of the preparations in our study, this is a potential scenario with other pharmaceutical preparations with diverse excipients. This highlights the need for further studies that explore other neutralization strategies and optimization protocols.

## Conclusion

Our findings underscore the critical need for comprehensive method suitability testing in microbial limit assessments of non-sterile pharmaceutical finished products, particularly when neutralization presents challenges. The detailed protocols we have developed not only facilitate the detection of microbial contaminants but also specifically address the often-neglected testing for the *B. cepacia* complex in aqueous formulations. This oversight in current QC practices highlights a significant gap in ensuring product safety, as many products are assumed to be adequately tested without further scrutiny. Further studies are needed to reinforce the safety of non-sterile pharmaceutical finished products from manufacturing to market, ultimately contributing to improved public health outcomes.

## Data Availability

The datasets used and/or analyzed during the current study are available from the corresponding author on reasonable request.

## References

[1] World Health Organization (WHO). WHO Expert Committee on Specifications for Pharmaceutical Preparations: fifty-eight report 2025. Available from: https://www.who.int/publications/i/item/9789240108264. Updated April 15, 2025; cited 2025 June 23, 2025.

[2] United States Pharmacopeia (USP). < Chapter 1111> Microbiological examination of nonsterile products: Acceptance criteria for pharmaceutical preparations and substances for pharmaceutical use. In: USP-NF Online. Rockville, MD: United States Pharmacopeial Convention 2013.10.31003/USPNF_M99830_01_01. Accessed 5 January 2024.

[3] United States Pharmacopeia (USP). < Chapter 61 > Microbiological examination of nonsterile products: Microbial enumeration tests. In: USP-NF Online. Rockville, MD: United States Pharmacopeial Convention 2013. 10.31003/USPNF_M98800_01_01. Accessed 5 January 2024.

[4] Jimenez L. Microbial limits. In L. Jiminez (Ed.), Microbial contamination control in the pharmaceutical industry. CRC Press; 1st ed., 2004. pp. 15-44. 10.1201/9780203026267.

[5] United States Pharmacopeia (USP). <62 > microbiological examination of nonsterile products: Tests for specified microorganisms. In: USP-NF Online. Rockville, MD: United States Pharmacopeial Convention 2013.10.31003/USPNF_M98802_01_01. Accessed 5 January 2024.

[6] Ratajczak M, et al. Microbiological quality of non-sterile pharmaceutical products. Saudi Pharm J. 2015;23(3):303–7. 10.1016/j.jsps.2014.11.015 PMC4475860 26106278

[7] Hawgood BJ.Prophylactic vaccination against cholera and bubonic plague in British India J Med Biogr. 2007;15(1):9–19. 10.1258/j.jmb.2007.05-59 17356724

[8] Glencross EJ. Pancreatin as a source of hospital-acquired salmonellosisBr Med J. 1972;2(5810):376–8. 10.1136/bmj.2.5810.376 PMC1788248 5063375

[9] Kallings LO, et al. Microbiological contamination of medical preparations.Acta Pharm Suec. 1966;3(3):219–28. 5967307

[10] Mugoyela V, et al. Microbial contamination of nonsterile pharmaceuticals in public hospital settings.Ther Clin Risk Manag. 2010;6:443–8. 10.2147/TCRM.S12253 PMC2952482 20957135

[11] Myemba DT, et al.Microbiological Quality of Selected Local and Imported Non-Sterile Pharmaceutical Products in Dar es Salaam, Tanzania. Infect Drug Resist. 2022;15:2021–34. 10.2147/IDR.S355331 PMC9038149 35480052

[12] Food and Drug Administration (FDA) Office of Regulatory Affairs. Pharmaceutical microbiology manual. 2020 . Accessed 6 June 2025. Available from: https://www.fda.gov/media/88801/download

[13] The GMP Journal. Microbiological Suitability Tests of Non-Sterile Preparations. 2022. Available from: https://www.gmp-journal.com/current-articles/details/microbiological-suitability-tests-of-non-sterile-preparations.html. Accessed 22 June 2025.

[14] Eissa ME, et al. Establishment of methods for microbial recovery: Miscellaneous non-sterile pharmaceutical dosage forms (Study I).Eur J Biomedical Pharm Sci. 2015;2(3):1272–81.

[15] Kumar SP, et al. Challenges and mitigation strategies associated with Burkholderia cepacia complex contamination in pharmaceutical manufacturing.Arch Microbiol. 2024;206(4):159. 10.1007/s00203-024-03921-9 38483625

[16] Tavares M, et al. Burkholderia cepacia Complex Bacteria: a Feared Contamination Risk in Water-Based Pharmaceutical Products.Clin Microbiol Rev. 2020;33(3). 10.1128/cmr.00139-19 10.1128/CMR.00139-19 PMC7194853 32295766

[17] Sutton S, et al. MICROBIOLOGY-A Review of Reported Recalls Involving Microbiological Control 2004-2011 with Emphasis on FDA Considerations of" Objectionable Organisms".Am Pharm Rev. 2012;15(1):42.

[18] Food and Drug Administration. FDA updates on 2017 *Burkholderia cepacia* contamination 2017 [Available from: https://www.fda.gov/drugs/drug-safety-and-availability/fda-updates-2017-burkholderia-cepacia-contamination

[19] El Chakhtoura NG, et al.A 17-year nationwide study of Burkholderia cepacia complex bloodstream infections among patients in the United States Veterans Health Administration. Clin Infect Dis. 2017;65(8):1327–34. 10.1093/cid/cix559 PMC5848224 29017247

[20] Mann T, et al. An outbreak of Burkholderia cenocepacia bacteremia in immunocompromised oncology patients.Infection. 2010;38:187–94. 10.1007/s15010-010-0017-0 20358245

[21] Coutinho CP, et al. Long-term colonization of the cystic fibrosis lung by Burkholderia cepacia complex bacteria: epidemiology, clonal variation, and genome-wide expression alterations.Front Cell Infect Microbiol. 2011;1:12. 10.3389/fcimb.2011.00012 PMC3417363 22919578

[22] Reik R, et al. Distribution of Burkholderia cepacia complex species among isolates recovered from persons with or without cystic fibrosis.J Clin Microbiol. 2005;43(6):2926–8. 10.1128/JCM.43.6.2926-2928.2005 PMC1151955 15956421

[23] United States Pharmacopeia (USP). General Chapter, 〈60〉 Microbiological examination of nonsterile products tests for *Burkholderia Cepacia* complex. Rockville, MD: USP-NF; 2019.

[24] Cundell A.M. Microbial monitoring of potable water and water for pharmaceutical Purposes. In L. Jimenez (Ed.), Microbial contamination control in the pharmaceutical industry (pp. 45-60). CRC Press; 2004.

[25] Food and Drug Administration (FDA). FDA advises drug manufacturers that *Burkholderia Cepacia* complex poses a contamination risk in non-sterile, water-based drug products. 2023. Available from: https://www.fda.gov/drugs/drug-safety-and-availability/fda-advises-drug-manufacturers-burkholderia-cepacia-complex-poses-contamination-risk-non-sterile#:~:text=The%20USP%20published%20a%20compendial,%2C%20USP%20). Accessed 22 June 2025.

[26] Devanga Ragupathi NK, et al. Accurate identification and epidemiological characterization of Burkholderia cepacia complex: an update.Ann Clin Microbiol Antimicrob. 2019;18:1–10. 10.1186/s12941-019-0306-0 PMC6360774 30717798

